# How lymphatic filariasis was eliminated from an urban poor setting in Santo Domingo, Dominican Republic

**DOI:** 10.1093/inthealth/ihy059

**Published:** 2018-10-04

**Authors:** Manuel Gonzales, Margaret C Baker, Ana Celestino, Danerys Santa Morillo, Amy Chambliss, Sarah Adams, Margaret Gyapong, Dominique Kyelem

**Affiliations:** 1 CENCET, Av. Juan Pablo Duarte, SD, Dominican Republic; 2 RTI International, 701 13th Street NW, Suite 750, Washington, DC, USA; 3 Universidad Autónoma de Santo Domingo, Ciudad Universitaria, SD, Dominican Republic; 4 Georgetown University, Department of Global Health, 3700 O St, NW, Washington, DC, USA; 5 George Mason University, 4400 University Drive, Fairfax, VA, USA; 6 University of Health and Allied Sciences, PMB 31, Ho, Ghana; 7 The Taskforce for Global Health, 330 West Ponce de Leon Ave, Decatur, Georgia, USA

**Keywords:** lymphatic filariasis, mass drug administration, neglected tropical diseases, urban

## Abstract

**Background:**

While progress has been made in the elimination of lymphatic filariasis, challenges that call for innovative approaches remain. Program challenges are increasingly observed in ‘hard-to-reach’ populations: urban dwellers, migrant populations, those living in insecurity, children who are out of school and areas where infrastructure is weak and education levels are low. ‘Business-as-usual’ approaches are unlikely to work. Tailored solutions are needed if elimination goals are to be reached. This article focuses on mass drug administrations (MDAs) in urban settings.

**Methods:**

We selected the urban poor area of Santo Domingo, Dominican Republic. With three rounds of MDA and with good coverage, elimination was achieved. We wanted to understand contributing factors to achieving good coverage. A qualitative study analyzed context, barriers and facilitators using a predefined framework based on review of the literature.

**Results:**

Results show that barriers commonly reported in urban settings were present (population density, lack of organization in household layout, population mobility, violence, shortage of human resources and challenges in monitoring treatment coverage). Tactics used included strong visibility in the community leading to high levels of awareness, the use of laminated photo sheets during house-to-house visits and a 1:4 supervision strategy. The importance of working through community leadership structures and building relationships with the community was evident.

**Discussion:**

The approach developed here has applications for large-scale treatment programs for lymphatic filariasis and other diseases in urban settings.

## Introduction

Lymphatic filariasis (LF) almost exclusively affects the poorest people worldwide and is one of the world’s most debilitating parasitic diseases. A total of about 120 million people are believed be infected with LF. Of these, about 40 million persons have either lymphedema (elephantiasis) or hydrocele (enlarged scrotum). A further 80 million, while often suffering from hidden internal damage to the renal and lymphatic systems, have no external signs of disease.^1,2^ Furthermore, LF causes mental health complications that reach far beyond its epidemiologic morbidity. In recent reviews of the association between LF, stigma and mental health,^3–5^ living with the disfigurement caused by LF has been associated in qualitative studies with stigma, despair, hopelessness, embarrassment, ridicule and frustration^6,7^ and with low quality of life in both the physical and psychological domains.^2,8–12^

LF was targeted for elimination by the World Health Assembly in 1997 (WHA50.29). The mass drug administration (MDA) strategy is to give single doses of effective treatment once a year to the entire population living in areas where the disease is endemic.^13^ Since 2000, a cumulative total of 6.7 billion doses of medicine have been delivered to almost 1 billion people and the success of this strategy has resulted in 20 countries no longer requiring MDA.^14^ However, the areas that are left to be treated are often those that are harder to treat, such as urban areas.^15^ Failure to reach such groups reduces a district’s treatment coverage (i.e. the percentage of persons swallowing the drugs out of the total population at risk).

The challenges of conducting MDA in urban areas include lack of a specific urban strategy, lack of health workers and volunteers, the presence of unorganized settlements and large numbers of migrants.^16^ The challenge of delivering MDA in urban settings is especially relevant given the continual increase in urban populations globally. As of 2015, 54% of the global population is urban and growing.^17^ One consequence of this rapid urbanization is that slums, termed the ‘urban poor’, are becoming home to an increasing number of the world’s population.^18,19^ While a recent article presents research showing that urban settings in West Africa may not even need to be treated,^20^ urban dwellers in other settings do require treatment and we need to know how to do that.

Although many counties have anecdotally reported the challenges of conducting MDA in urban settings, an MDA program in the urban poor part of Santo Domingo, the capital city of the Dominican Republic, reported good coverage over 3 y. The objective of this study is to present a qualitative analysis of this ‘positive deviant’ and increase our understanding of what can be successful in urban settings. Because the drivers of compliance are partially generalizable, our findings may also have potential application beyond lymphatic filariasis campaigns, including preventive chemotherapy (PCT) campaigns for other neglected tropical diseases (NTDs) and community-based treatment programs for tuberculosis, human immunodeficiency virus (HIV), childhood pneumonia and diarrheal disease.

## Materials and methods

The study of ‘positive deviants’ was first conducted in the field of nutrition,^21^ based on the observation that there are communities or individuals whose behaviors or strategies enable them to find solutions to a problem that others are struggling with, and has been used in social and behavioral studies since then. Qualitative methods were used to investigate the MDA program in Santo Domingo, resulting in a rich description of the case and proposed strategies to be tested in other settings.

### Data collection

Data collection included both review of program documents and interviews with key informants. Semistructured in-depth interviews and focus group discussions (FGDs) were conducted with participants representing six different perspectives, selected to represent all the key players involved in the design and delivery of the MDA: community members who were passive recipients of the MDA intervention, community volunteers who acted as drug distributors and supervisors, leaders of community organizations and program managers from both the ministry’s LF program and the nongovernmental organization (NGO) involved in the MDA Centro Juan Montalvo (Table 1). Data from these initial interviews were reviewed and it was determined that the saturation point was reached and no further interviews were necessary. A total of 85 persons were interviewed.

**Table 1.** Study participants

Type of participant	Instrument used	Total number of participants
Community recipients of MDA	3 FGDs	30
Community drug distributors and supervisors	3 FGDs	33
Community leaders	1 FGD	10
LF program staff	1 FGD	8
LF program directors	2 interviews	2
NGO program managers	2 interviews	2

All program managers (current and previous) who had been involved in the MDAs, except one who had left the country, were included in the study. All high-level community leaders were included in the community leaders FGD. Drug distributors, supervisors and community members were selected by the community leaders as persons who would be knowledgeable about the MDAs.

Semistructured interviews and FGD guides were structured around the following themes: contextual information on the community; knowledge of LF; description and perceptions of the MDA program, including the organizations involved; and barriers and incentives to participation in MDAs. Interviews and FGDs lasted between 1 and 1.5 h. Question guides were translated into Spanish and interviewers were trained in the use of the guides.

### Ethics

This study was approved by the director of CENCET in the Dominican Republic and IRB approval was obtained from Georgetown University—IRB Number: 2011-168. Informed consent was obtained in writing from all participants.

### Data analysis

We recorded all interviews and transcribed them verbatim, storing and organizing them in MAXQDA version 10 (1989–2013; VERBI Software, Berlin, Germany). ACh coded the data using both predefined themes constructed from literature reviews and topic guides as well as new codes created based on topics that emerged during coding as per the grounded theory approach. MCB then read all coded data, comparing across participant types, creating an initial list of theoretical propositions. SA reviewed all transcripts to test these propositions looking for information that would either contradict or support theories, based on the rival theory explanation proposed by Yin.^22^

Findings are presented using the framework presented in Table 2, with reference to the NTD logic model created by MCB and presented in an article by Lemoine et al.,^23^ an economic framework developed by Weaver^24^ and supported by evidence presented in a literature review conducted by Krentel et al.^16^

**Table 2.** Framework for understanding the factors influencing treatment coverage obtained by LF MDAs

**Recipient’s willingness to comply—knowledge, attitudes and beliefs**
Knowledge of disease—although in-depth knowledge of the disease has not been found to be an important predictor of compliance, some knowledge of LF, especially of mosquitoes as transmitters, and that MDAs are to prevent LF are positive predictors of MDA participation. Perception of risk. Perception of benefits of taking part in MDAs, including deworming benefits. Awareness of the program and knowledge of the elimination strategy. Experiences during previous MDAs. Dislike of normal minor side effects. Rumors about negative side effects of drugs, including that they cause sterility. General dislike for modern medicine. Lack of trust in drugs (poor quality, too many, too big). Peer pressure to participate. Trust in persons delivering the MDA—affected by whether they are known, from the same area, from the same caste and their status in the community (e.g. respected health worker).
**Recipient’s capacity to respond**
Community organizations utilized to help mobilize the MDA.
**Program delivery and monitoring** Quality of supervision. Monitoring ingestion of medication through directly observed MDA. Availability of human resources, often increased by using community volunteers as drug distributors. Knowledge and skills of the drug distributors. Quality training. Motivation of drug distributors and health staff. Logistics—drug and materials supply, timing of MDAs, availability of funding. Inability to monitor treatment coverage (poor numerators and/or denominators).

## Results

### Context and challenges

**Figure 1.**
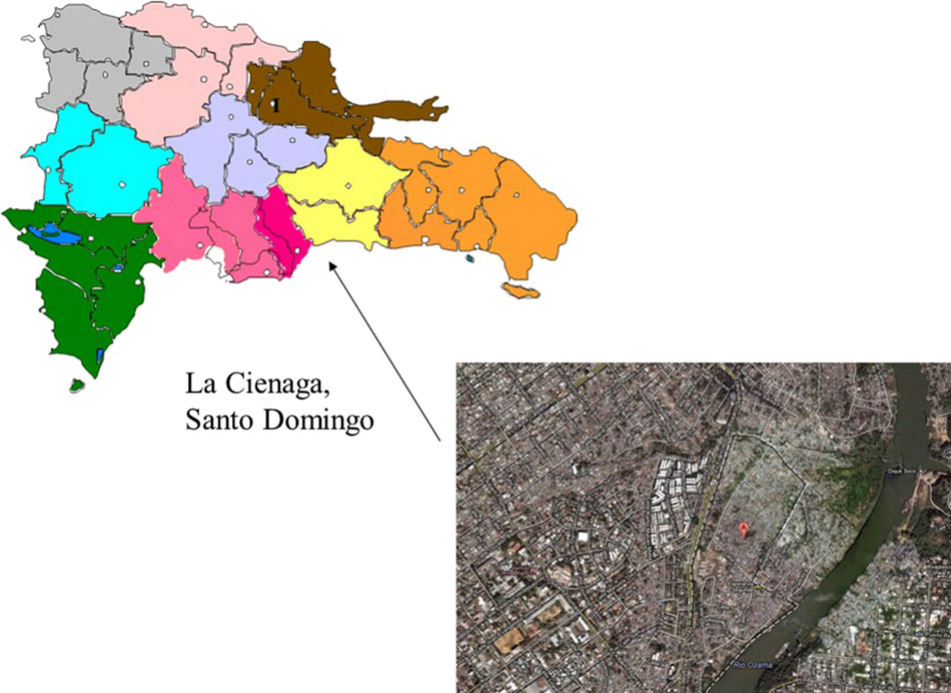
Map showing the location of the study site—La Ciénaga, Santo Domingo, Dominican Republic. Image captured from Google Maps in 2011.

This research is set in an urban poor sector of Santo Domingo, Dominican Republic, divided into three sectors—Los Guandules, Guachupita and La Ciénaga—with a total population of 50 000 (Figure 1). This setting has many challenges in common with other urban poor areas around the world. The population is young, migrants from rural areas, part-time and informal workers, dependent on a cash economy, living in substandard housing and with limited access to services including water and sanitation, education and health.

MDAs were implemented in 2004–2006, which was a period of change for the community. Between 2002 and 2012, improvements were made to the physical environment and to the services provided, including the addition of a fire station, a new school, paved roads, improved housing, more students going to university, regular trash collection, increased police presence, more and better primary care health facilities, a reduction in the number of mosquitoes and covered drains.

However, although much has improved, this area remains poor and the issue of violence remains foremost on people’s minds. Community members in this study noted that the nature of the violence has changed—while there seemed to be some agreement that the number of killings had decreased, people reported that there are more firearms used today and that gang members were getting younger. Some residents reported that they feel less secure today because the violence is more random.

The sociogeographic nature of the setting provided additional barriers to implementing MDAs. Both the population density and lack of organization and planning in the layout of the houses posed a challenge to program organizers, especially in the first year. Houses were not organized into neat grids, there were many narrow alleys that were not accessible by car, and steep stairs connected different zones. External program coordinators reported often getting lost; in fact, community members themselves reported getting lost if they were in a part of the community where they did not live. Population mobility was another challenge, with many persons out working during the day. Safety was an important consideration in this area associated with drugs, gangs, police corruption and increased use of firearms; several of the LF program team noted that they had been afraid to work in this area. Traditional human resources for health care were limited in this area. Monitoring treatment coverage also posed a challenge. Many people from outside of the target population had heard about the campaign and came demanding treatment (note that in urban areas, people may live on the other side of a road from the target population). Recording those persons as treated increased the numerator and thereby inflated the reported coverage figures. Accurate census information was also missing, making it difficult to estimate treatment coverage.

Community members and their leaders report that the first LF MDA in this community came at a time when the community was working on an agenda to address the many social problems that it faced. It had organized itself into coordinating bodies, the coordinators of which are referred to in this article as ‘community leaders’. Project managers and community members commented on the community’s involvement and active participation in the implementation of several different projects (see quote in Table 3).

**Table 3.** Illustrative study participant quotes

Recipient’s willingness to comply—knowledge, attitudes and beliefs
**Strong awareness campaign and high program visibility** *‘…the leaflet was used to access people immediately. We would present ourselves and explain why we were there and then we would show them the graphics and the effects that* [the disease or infection] *caused…one did not* [show] *it at the start so that the person would not get a fright…One would give them the explanation and at the end, as a form of persuasion, to convince them, the graphic would be shown. And so the persons accepted’.* FGD drug distributors, Los Guandules ‘When I saw that they showed me the pictures of a man who had his.. his parts, the testicles of that size, I said: “wow”, because I had not wanted to take them (the tablets),…when they showed me that I said: “shit”…that was the means by which I was convinced to take them’. FGD, Guachupita ‘…one would talk to them and explain well what the disease was, then at a given moment one would show them the photos and they would tell you: look at that, it’s because of this disease that we have to take the tablets, because if you do not this can happen, when they saw the testicles they would say: ayy! No, give me the tablets because I am going to take them, I am going to take them’. FGD community leaders
**Build trust** *‘…I refused* [the idea of taking the drugs] *because I saw others who would get dizzy and feel bad…but then there were people whom I trusted who were not going to tell you one thing when it was the other, who were the people who were distributing the drugs’.* FGD community leaders, La Ciénaga *‘…for someone that I do not know to come to my house, there were two of them who came to treat me, and I said no, but the supervisor I know, I know (s)he is not going to tell me lies…we have known each other for a long time…*[she] *tells me it is to treat this disease and there one engages, but someone that I do not know…’* FGD drug distributors, Guachupita ‘…I had the experience of some young people, because some young people were chosen to medicate,…that were youth from the barrios who were not community leaders, and there were people who would not allow themselves to be treated because there was no trust, but once a community leader went, they would see a community leader directing the process, they would say: “no problem”. With the simple act of seeing a community leader, there was no need to give much explanation’. FGD community leaders
**Leverage social capital** *‘It was through the Centro Montalvo that we had contact with all the* [community] *organizations, and from there to their leaders, and the leaders made everything easy for us. For us everything was made very easy working through the leaders—they called together the people, we would meet with the people and train…so everything was agreed with the leaders and with the* [community] *members there was great participation, it was very open, for me it is one of the most open processes that I have seen.* CENCET manager ‘[leading to success was] *having the institutions involved. There were some we already knew, that was very important, the knowing, because for example we knew the Centro Juan Montalvo, some of us knew CENCET, others knew the community based organization, we belong to the community…that was primordial.’* FG Community Leaders

Recipient’s capacity to respond
‘…this community characterizes itself by always wanting to know how they can be involved in projects, how they can help and how the project will help, and it does not matter whether there is an incentive given, if not even five pesos are given, because what is more important is the benefits that can be left for the barrio, the communal benefits’. FGD community members, La Ciénaga ‘We always adopt the methods of having the community integrated in the whole process, because that is how they really feel an affinity for the problem and they also collaborate with the solution to the problem, because the problem in basically theirs…’ CENCET member

Program delivery and monitoring
*‘The people* [drug distributors] *would go to a house, if that person refused to take the tablet, they would not push the issue, they would just say “it’s ok. Thank you for your time”. Then after the supervisor would go to the house to speak with that person, and try to explain better to see if the person could be persuaded and medicated’.* FGD community member, Guachupita *‘The CENCET people have that gift of getting close to people, they are not imposing, that is to say they consulted with the community, they never imposed…and of course* [the community members] *would take on the leadership…the leaders of those organizations were already very involved in the process’.* Centro Juan Montalvo manager *Participant 1: ‘They* [CENCET] *saw themselves as if they were from the community, they integrated very well with us, we were like one, it was as if they were used to the barrio, they came’.* Participant 2: ‘They were as if they were from here’. Participant 3: ‘It was as if they were from here. Sometimes one would see them walking about the barrio, one felt an affinity with them, and they would speak very well with us, very genuine, and they would explain things well, any question one had they would answer it…they would reply with that enthusiasm of wanting other to learn how to do things’. FGD drug distributors, La Ciénaga *‘They* [CENCET] *put a lot of confidence in us, but they also supervised us, they never left us alone. There was a permanent team from CENCET* [at the school campaign base] *and* [the CENCET director] *was in constant communication by cell phone. He put a lot of confidence in us, but he was monitoring the process’.* FGD community leaders

In the research described here, the community reported that prior to the LF program they had not heard of LF (although there were some reports of at least one individual with lymphedema in the community). There was no history of MDA campaigns within the community and, while one of the drugs used—albendazole—was known to many, the majority had not heard of diethylcarbamazine citrate. Fear of drugs was an issue. The idea of swallowing several of these unknown tablets was reportedly initially frightening. Both drug distributors and community members reported that on first contact there were a large number of persons refusing drugs for various reasons, including fear of side effects, fear that the drugs were poisoning people, fear that the drugs would cause sterility in women and the belief that they were not at risk, especially among the men. Minor side effects were common, with many persons reporting feeling sleepy or dizzy.

### Program impact

Because of the proximity to the river and the poor socioeconomic conditions of the community, it was determined that this population was at risk for LF. In 2002, the government organization Centro de Control de Enfermedades Tropicales (CENCET) conducted a survey by taking a blood sample from a finger prick. They found that 10.7% of those tested had filarial antigens in their blood (antigenemia rate) and in 2.5% of survey participants the parasite could be seen in their blood smear under a microscope (microfilarial rate). Eight years after the last MDA, a transmission assessment survey (TAS) was conducted. Of 815 children between 5 and 9 y old tested by immunochromatographic test (ICT), zero (0.0%) were positive. This indicated that the area ‘passed’ the TAS and that transmission has been interrupted in this area.^25^ Elimination after three rounds of MDA is congruent with mathematical model predictions given the high treatment coverage observed.^26^

### Program outcomes

Treatment coverage rates, defined as the total number of persons treated as a percentage of the total population living in the target area, were reported as 67%, 92% and 86% for 2004, 2005 and 2006, respectively (CENCET data). The population was counted in a pre-MDA census conducted in 2006 by the program and applied retrospectively to previous years.

Although a post-MDA coverage survey was conducted in 2004 to validate reported coverage, these data were lost, highlighting the importance of the database being developed by the World Health Organization to store and manage NTD data.

### Program implementation

#### Health education and community sensitization

Overcoming knowledge barriers was seen as a process—the first year was the hardest and the second and third years became considerably easier as the community became familiar with the program.

At the time of this study (following three rounds of MDA) the community did not seem to understand the disease in any detail but was very clear in its comprehension of two key points: the fact that filariasis is transmitted by mosquitoes (of which there is a great abundance in this community) and the symptoms of the disease. The phrase ‘it affects the hanging parts’ to describe the disease, coined by the CENCET team and used in its advocacy materials, had obviously stuck with the community, as evidenced in the FGDs.

Multiple communication channels were employed to reach the community, presenting relevant information on the disease and the MDA strategy. Study participants reported obtaining information via one of the many small community-based meetings (*charlas*), loudspeaker announcements from trucks and announcements in schools and churches. Word of mouth was also identified as an important source of information. Television was used, but was mentioned by only a couple of study participants.

The one-to-one (or one-to-family) health education given by the drug distributors in person as they went house to house during the MDA was identified by study participants as being crucial in terms of persuading people to take the drugs being offered. A tool used during this interaction was a laminated card depicting photos of persons with lymphedema on one side and hydrocele on the other. These were emphasized many times by the majority of participants as a key to success. This strategy was reported to be especially useful in convincing the men, who were the ones initially less receptive to the idea of taking the medication offered (see quotes in Table 3).

The researchers who conducted the interviews and FGDs noted that as they were talking with the study participants they were struck by the high visibility of the MDA campaign and of the ‘buzz’ present in the streets during the MDA. During the intense weekend period of the campaign there were 320 drug distributors plus 64 supervisors in the streets, all wearing T-shirts and caps in a distinctive bright green color (intentionally changed from white after the first MDA to make them more visible) and carrying identification cards with the official public health logo. During the MDA, people would talk to each other about having taken the medication, causing others to stand at the entryway of alleys to ensure that the drug distributors would not miss them.

#### Strategies for dealing with side effects

Strong emphasis was placed on the management of side effects. During training, all drug distributors had to take the medication themselves in order to experience first-hand the types of side effects that the drugs could cause and to give them confidence in the safety of the drugs. Emphasis was also placed on involving doctors at the local health centers during the first MDA, and health centers were kept open over the weekend with doctors being paid an incentive for overtime and given free drugs for dealing with minor side effects. Immediate follow-up was given to persons complaining of side effects during the MDA, helping to avoid the spread of misinformation.

#### The importance of trust

This research highlights the important role that ‘trust’ played in the MDA—trust between the community and the government, trust between the community and its leaders and trust between the community and the drug distributors. In this research, facilitating the trust of the community in the drug distributor was achieved using a number of different strategies: 1) recruitment by the community of persons to treat those who lived close to their own homes (belonging to the same group), 2) issuing official identity cards, T-shirts and hats (role-based trust), 3) investing in training so that they came across as knowledgeable and professional (role-based trust) and 4) providing supervisory support by a more senior community volunteer in a 1 supervisor:4 drug distributors ratio (presence of a third party) (see quotes in Table 3).

**Figure 2.**
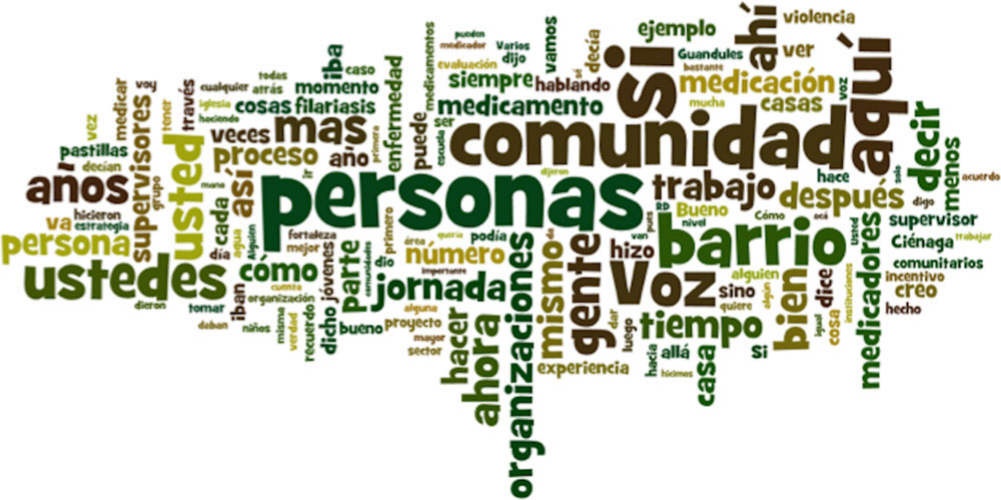
Illustration of the most utilized words that appear in the transcripts.

We also observed the importance of the national LF program’s ability to harness and use social capital. A strong factor in the success of this program was the ability of the CENCET team to leverage resources available within the community. The fact that the community was so central to this intervention is illustrated by an analysis of the number of times words appear in the transcripts, with the words *community*, *people* and *barrio* being some of the most utilized words (Figure 2). This program was seen by CENCET as a program for the community to be implemented by the community (see quote in Table 3).

The ability of CENCET to leverage these community resources was facilitated by the Centro Juan Montalvo, a local NGO that has been working closely with the community and its leaders over a number of years on various social improvement activities. The Centro Juan Montalvo is respected and well known by the community and served as a broker, paving the way for a strong relationship between CENCET and the community. While one study participant questioned whether CENCET was not already well enough known in the community and noted that it cost extra to bring in a third party, all others highlighted the importance of this NGO’s role (see quotes in Table 3).

While the LF program leveraged existing social capital in the community, working with the community also built the community capacity further. Community leaders reported that through participation in the MDA they got to know their community better and saw sectors they had not known existed. They also reported that they had used MDA management tools to later organize other projects, such as the campaign against violence.

#### Access

Access to health care can be defined in terms of cost of treatment and proximity to obtain it. In the global program for elimination of LF, the drugs are offered free to the end user—thanks to a strong public–private partnership with large-scale drug donations from GlaxoSmithKline. The delivery strategy used was house to house plus fixed posts. Because the community in this urban setting is often not at home during the week, the campaign was run intensively on weekends with follow-up during the week for persons who had not been home.

### Capacity of drug distributors and supervisors

#### Training

The CENCET team noted the importance that they placed on achieving quality training by setting the maximum number of persons per training session at 30, having a CENCET team member do the training, using interactive pedagogical methods and repetition of learning—key messages were repeated before the MDA every year, with refresher trainings given by team supervisors the day before the MDA and again on the morning of the MDA. Supervisors received more training than the drug distributors.

Emphasis was put on developing professionalism in the drug distributors; for example, one of the community members described the drug distributors as ‘professional’ because they did not touch the tablets with their hands.

Drug distributors often referred to the training as ‘very cool…with a lot of detail’ and as having provided them with confidence to do their job. Morale appeared to be high, with many volunteers, from community leaders to drug distributors, reporting that the MDA was a very nice experience and that they had enjoyed taking part.

#### Supervision model

There were various levels of supervision. The CENCET team together with the Centro Juan Montalvo provided the highest level of supervision, where the style was one of ‘being present’, supporting and providing assistance. The community leaders were responsible for coordinating and supervising zones and there was a volunteer supervisor for every four volunteer drug distributors.

It is this bottom level of supervisors, with a 1:4 ratio, that was identified as a novel strategy and the key to success in this study. The role of these supervisors was to provide reinforcement to the training that drug distributors received, to check that all houses were visited, to follow up on persons not at home and to conduct regular spot checks of drug distributors using a checklist instrument provided by CENCET. The checklist included checking the attitude of the drug distributor, that they were providing the correct dosages and that no medication had been left behind for persons to take later. Drug distributors would also frequently report persons who did not want to take the treatment to their supervisors. The supervisors would then follow up and take some time to talk with these persons in an attempt to persuade them to take the treatment.

The program used maps available from the community organizations to assign work to the drug distribution teams. While the maps were not precisely accurate, they did allow sectors to be divided between teams. Having supervisors work in their own neighborhood also facilitated the supervisor’s monitoring of program coverage.

Finally, there was also considerable emphasis placed on observing people take the medication, with supervisors conducting regular checks that this policy was being followed. Both drug distributors and community participants were aware of this policy and noted that it was followed.

#### Monitoring and evaluation

Before the third MDA, a detailed census was conducted. This activity took 2 weeks and was conducted by the MDA team supervisors with the support of some drug distributors. Standard census methodology was used. The CENCET LF director had experience in conducting censuses. There were three important benefits of the census: first, accurate denominator data were obtained and used retrospectively to adjust reported coverage rates; second, the team supervisors had a clear and detailed understanding of the geography of the area they were responsible for medicating; and finally, the census was an ideal time to verbally pass on information of the upcoming MDA.

The problem of inflated numerators due to treating persons outside the target population was addressed by training drug distributors to record the treatment of such persons using a separate form.

#### Strong program management

The program benefitted from a strong team, which valued supportive supervision (see quotes in Table 3).

## Discussion

The challenge that we address in this article is how to obtain high treatment coverage in urban settings. Building on the framework for understanding predictors of treatment coverage outlined in Table 2, we highlight a significant number of barriers to delivering and monitoring an effective program that exist in an urban setting and identify strategies that were used to overcome them. We show that in this urban setting the usual barriers to an individual’s willingness to comply (Table 2) exist and suggest that a more finely tuned strategy may be needed to overcome these barriers. We also note the importance of addressing a community’s capacity to respond by leveraging existing social networks.

### Overcoming multiple barriers, some specific to urban poor settings

While many of the barriers to MDA compliance described in the literature are also found in this study (Table 2), we found additional barriers related to delivering and monitoring a program effectively in an urban setting: population density and lack of organization in household layout, population mobility, violence, a shortage of human resources and challenges in obtaining accurate numerators as well as denominators for monitoring treatment coverage.

Specific strategies used to address those particular challenges included working through community organizations to identify volunteer drug distributors; assigning drug distributors to treat houses in their own immediate neighborhood where they are known and are familiar with the local geography; conducting a pre-MDA census; using registers that record separately the treatment of persons from outside the target group; marking houses that have been treated with a sticker, allowing easy identification by supervisors for those who required follow-up and timing MDA distribution on the weekend when people were more likely to be at home. A summary of the methods used to overcome the barriers found is presented in Table 4.

**Table 4.** Summary of strategies and activities used to obtain good coverage in this urban MDA

Strategy	Activities
Strong awareness campaign and high program visibility	Multiple communication channels
	Clear messaging, e.g. ‘disease that affects the hanging parts’
	House-to-house health education during MDA
	Laminated photo sheets
	About 400 persons in streets during weekend MDA, wearing distinctive program T-shirts and caps
	Peer-to-peer communication as MDA was ‘talk of the town’
Manage side effects proactively	All drug distributors and supervisors take medicine during training giving experience and building confidence
	Local doctors trained and health centers open during the weekend to address side effects
	Free drugs for managing side effects
	Immediate response by supervisors to all persons with side effects
Build trust	Recruitment in the community of drug distributors who treat those close to their own homes where they are known
	Drug distributors given official identity cards and uniforms (T-shirts and caps)
	Investment in training drug distributors to be professional
	Supervision by more senior, and better known, community members
Leverage social capital	Used community structure and existing leadership
	Community designed and led MDA, including the selection of drug distributors and first-level supervisors
	Use of an NGO as an intermediary that is already known to the community
Easy access	Free treatment
	Combined use of house-to-house and fixed posts
	Weekend based when people not at work
	Follow-up weekday and evenings
Build strong capacity in drug distributors with quality supervision	Quality training—maximum of 30 persons, national program trainers, interactive pedagogical methods and repetition of learning
	Ratio of one first-level community supervisor to four drug distributors
	Use local maps to clearly assign treatment and supervision areas
	Directly observed treatment is closely monitored
Adapted monitoring	Pre-MDA census led by a person with training in how to do a census
	Recording of external population treated on separate tally forms
	Post-MDA coverage survey
Strategic use of available resources	Use of a national team to lead urban MDA

Challenges assessed in this study were also found in studies conducted in Ghana, India and Kenya, where there was also a lack of prior awareness of MDA, a lack of knowledge about LF and a lack of perceived risk among community members.^27–29^ Additional challenges included unavailability of funding, especially for identification materials for drug distributors, and a lack of motivation and inadequate remuneration for drug distributors.

### Innovative strategies to obtain quality

Quality in training drug distributors was also emphasized, together with a strong supervision strategy. In this regard, this study identifies two innovative strategies that should be considered for wider use in the global program, especially in settings such as urban environments, where a high-quality program is required: the laminated picture sheets and the 1:4 supervision strategy described above.

### Implications for resources

There is no evidence that the Dominican Republic’s program had significantly more funds to implement an MDA than other countries. While analyzing the cost of treatment in La Ciénaga was outside the scope of this study, data on costs at the national level have been published showing the financial cost per person treated in the Dominican Republic is US$0.87, similar to that of other comparable countries, US$1.00 in Egypt and US$1.96 in Haiti.^30^

On the other hand, the Dominican Republic’s national LF program was probably better off than many other LF programs in terms of human resources at the national level. The national team was comprised of nine persons employed full time to manage a program for an at-risk population of 1.3 million. This included a coordinator, accountant, driver, secretary and six program facilitators. The salary of this team was initially paid with external funding from the Bill and Melinda Gates Foundation and in 2006 was subsumed by the Ministry of Health. This team was mobilized in its entirety to plan and implement the MDA in La Ciénaga, conducting the MDA at a different time of year from those in other focal points.

Of course, additional attention to quality will require more resources—both financial and human. But front-loading can pay off. With low treatment coverage, many more rounds of MDA will be required, with the danger of fatigue for both target groups and donors.

### Importance of trust

The literature on trust generally accepts that trust is fundamentally a psychological state where ‘one not only thinks trust but feels trust’, with predictors of trust including positive past experiences, the presence of a third party that is trustworthy, a sense of belonging to the same group or social organization and role-based trust.^31,32^ This case highlights the important role that ‘trust’ played in the MDA—trust between the community and the government, trust between the community and its leaders and trust between the community and the drug distributors. The importance of trust has also been emphasized by others in relation to MDAs,^16^ as well as in communication with parents about childhood vaccinations^33^ and in using community volunteers to do home visits for adherence to long-term antiretroviral treatment for HIV.^34^

### Leveraging social capital

Related to the concept of trust is the notion of social capital, used to refer to the existence of resources available due to the strength of relationships between community members based on social trust, reciprocity and civic engagement.^35^ The theory is that public health programs can maximize their success by leveraging existing social capital or creating new social capital. In the study presented here we note the importance of community members’ willingness to volunteer their time based on their perception of how it would benefit their community and of having an existing structure (albeit in its infancy) for organizing community development activities.

In summary, two guiding principles are identified for implementing urban MDA: ensure quality in each element of the MDA (communication, training, supervision) and assess the social network to identify how to gain the communities’ trust. These elements have also been identified as key in studies of urban MDAs in Pondicherry, India^27^ and Freetown, Sierra Leone.^36^ A list of strategies that can be used in urban settings is presented in a table from which other programs can pick and choose depending on their situation and level of resources.

### Study limitations

While this study provides some guidance for other urban MDAs, it is limited to a poor urban population. One of the challenges in urban settings is the heterogeneity of the population, with persons of a much higher socioeconomic status living in large compounds surrounded by fences and guarded by dogs providing a different set of challenges. Other studies will be needed to address challenges more specific to those and other urban populations.

## 

**Authors’ contributions:** The study was designed by MGo, MCB, MGy and DK. Interviews were conducted by ACe and DSM, two local researchers previously unfamiliar with the LF program but with many years of experience conducting qualitative research in our target population. Prior training on LF and explanation of the study guides was provided by MCB and MGo. MGo was the program director and MCB participated in all three MDAs as an external observer. MCB served as the principal investigator on the project and MGo coordinated the field work with ACe and DSM and reviewed drafts of the manuscript. ACh and SA were responsible for the analysis and interpretation of data. ACh coded the data using both predefined themes constructed from literature reviews and topic guides and new codes created based on topics that emerged during coding, as per the grounded theory approach. MCB then read all coded data, comparing across participant types, creating an initial list of theoretical propositions. SA reviewed all transcripts to test these propositions, looking for information that would either contradict or support theories. MGo, MCB, ACe, DSM, ACh, SA and MGy read and approved the final version of the article.

**Acknowledgements:** We dedicate this article to the late Dr. Dominique Kyelem. It was his vision that enabled this study, and others like it on implementing MDAs in urban settings, to be carried out. He was hard working, intelligent and kind. We remember him with gratitude for all that he was and did. We also acknowledge with thanks Kent Weaver and John Kraemer, professors at Georgetown University, for their continued encouragement and reviews of several drafts. We also thank Anna Geduldig, Kirsty Sievwright and Lisa Ghaffari, students at Georgetown University, for their assistance with reviewing the literature and editing. Finally, we thank everyone who agreed to be interviewed as part of this study.

**Funding:** This research was supported in part by funds from the LF Support Center at The Taskforce for Global Health as part of a grant (43922) from the Bill and Melinda Gates Foundation focused on ‘Resolving the Critical Challenges Now Facing the Global Program to Eliminate Lymphatic Filariasis’. The funders had no role in the study design, data collection and analysis, decision to publish or preparation of the manuscript.

**Competing interests:** None declared.

**Ethical approval:** This study was approved by the director of CENCET in the Dominican Republic, and institutional review board approval was obtained from Georgetown University (2011-168). Informed consent was obtained in writing from all participants.
